# Cell Wall-Associated Virulence Factors Contribute to Increased Resilience of Old *Cryptococcus neoformans* Cells

**DOI:** 10.3389/fmicb.2019.02513

**Published:** 2019-11-07

**Authors:** Erika P. Orner, Somanon Bhattacharya, Klea Kalenja, Danielle Hayden, Maurizio Del Poeta, Bettina C. Fries

**Affiliations:** ^1^Department of Microbiology and Immunology, Stony Brook University, Stony Brook, NY, United States; ^2^Department of Medicine, Division of Infectious Disease, Stony Brook University, Stony Brook, NY, United States; ^3^Northport Veterans Affairs Medical Center, Northport, NY, United States

**Keywords:** *Cryptococcus neoformans*, aging, virulence, antiphagocytic protein, cell wall, melanin

## Abstract

As *Cryptococcus neoformans* mother cells generationally age, their cell walls become thicker and cell-wall associated virulence factors are upregulated. Antiphagocytic protein 1 (App1), and laccase enzymes (Lac1 and Lac2) are virulence factors known to contribute to virulence of *C. neoformans* during infection through inhibition of phagocytic uptake and melanization. Here we show that these cell-wall-associated proteins are not only significantly upregulated in old *C. neoformans* cells, but also that their upregulation likely contributes to the increased resistance to antifungal and host-mediated killing during infection and to the subsequent accumulation of old cells. We found that old cells melanize to a greater extent than younger cells and as a consequence, old melanized cells are more resistant to killing by amphotericin B compared to young melanized cells. A decrease in melanization of old *lac*Δ mutants lead to a decrease in old-cell resilience, indicating that age-related melanization is contributing to the overall resilience of older cells and is being mediated by laccase genes. Additionally, we found that older cells are more resistant to macrophage phagocytosis, but this resistance is lost when *APP1* is knocked out, indicating that upregulation of *APP1* in older cells is in part responsible for their increased resistance to phagocytosis by macrophages. Finally, infections with old cells in the *Galleria mellonella* model support our conclusions, as loss of the *APP1, LAC1*, and *LAC2* gene ablates the enhanced virulence of old cells, indicating their importance in age-dependent resilience.

## Introduction

For a pathogen to survive in a host during infection, it must be able to sense its environment and respond accordingly. In the human host, fungal pathogens can withstand high body temperatures, changes in pH and nutrient composition, and the host response by modifying their cell wall. *Cryptococcus neoformans* is a ubiquitous environmental fungus that causes disease in humans who are immune compromised. *C. neoformans* is responsible for upward of 15% of AIDS-related deaths worldwide (Rajasingham et al., 2017). During infection, alveolar macrophages are the first line of defense against *Cryptococcus* (Alvarez and Casadevall, 2006). In order to establish an infection, *C. neoformans* must find a way to inhibit macrophage phagocytosis and phagocytic killing. *C. neoformans* employs a number of virulence mechanisms to combat macrophage attack including age-dependent cell wall modification (Bouklas et al., 2013), melanization, and secretion of the antiphagocytic protein 1, App1 (Del Poeta, 2004).

Previously, our lab has shown that generational aging of fungi contributes to enhanced resilience in the host (Bouklas et al., 2013, 2017a,b; Bhattacharya and Fries, 2018; Bhattacharya et al., 2019; Orner et al., 2019). *C. neoformans*, like other yeasts, undergoes asymmetric division when replicating, resulting in a mother cell’s phenotype that continuously evolves with each division (Bouklas et al., 2013). Generationally aged cells (i.e., 10-generation-old cells) show an increased resistance to phagocytic ingestion, phagocytic killing, and even antifungal killing (Bouklas et al., 2013, 2017a; Orner et al., 2019). What mediates this age-dependent resilience is not known, but experimental data demonstrates the selection and accumulation of generationally older cells during infection in rats and in humans (Bouklas et al., 2013, 2017a).

Antiphagocytic protein 1 (App1) is a virulence factor that is unique to *C. neoformans* (Luberto et al., 2003) and located in the cell wall of *C. neoformans* (Qureshi et al., 2012). This protein is also secreted into the supernatant of cultures and detectable in bronchoalveolar lavage fluid, serum, and cerebral spinal fluid of patients (Luberto et al., 2003; Stano et al., 2009; Williams and Del Poeta, 2011). App1 inhibits phagocytosis by macrophages through a complement-mediated mechanism where the App1 protein competes with iC3b for binding to complement receptor (CR) 3 on macrophages (Stano et al., 2009). During infection, iC3b opsonizes microbes and binds to complement receptor 3 on professional phagocytes like monocytes, macrophages, and dendritic cells to aid in phagocytosis (Stuart, 2002). When App1 binds to CR3, it reduces attachment and ingestion of *C. neoformans* into macrophages both *ex vivo* and *in vitro* in a dose-dependent manor (Luberto et al., 2003). Knockout mutants lacking *APP1* are less virulent in mice, indicating this virulence factor plays an important role in establishing infection. Interestingly, Qureshi et al. (2012) found App1 to have amyloid properties and argue it may also play additional roles in pathogenesis. For example, amyloids have been shown to help evade the immune system by producing a protective coating around the cell wall in various other microbes (Gebbink et al., 2005; Qureshi et al., 2012). Furthermore, different amyloids have been shown to be important for melanin biosynthesis (Qureshi et al., 2012).

Melanin production is a key virulence factor for a wide variety of microbes and multicellular organisms including fungi, bacteria, plants, and animals (Howard and Valent, 1996; van Duin et al., 2002; Nosanchuk and Casadevall, 2003). Melanin synthesis occurs in the cell wall through the oxidation of phenolic substances like dopamine, epinephrine, and norepinephrine into quinones which then polymerize into pigmented melanin products (Williamson, 1994). These substances are found in high concentrations in the central nervous system and may contribute to *C. neoformans’* tropism for the central nervous system (Polacheck et al., 1982). Melanization contributes to resistance against antibody-mediated phagocytosis and phagocytic killing by macrophages (Wang et al., 1995; Casadevall and Perfect, 1998; Zhu and Williamson, 2004) and resistance against free-radical killing by reactive oxygen and nitrogen species (Wang et al., 1995; Missall et al., 2004). Furthermore, melanization provides protection against antifungals like amphotericin B, the first line therapeutic against *C. neoformans* (van Duin et al., 2002).

The laccase gene, *LAC1* encodes the rate-limiting enzyme that catalyzes polymerization of quinones and has been the focus of most *C. neoformans* melanization studies (Torres-Guererro and Edman, 1994; Williamson, 1994). *LAC2* is another cryptococcal laccase gene that exhibits 72% amino acid homology to *LAC1* (Missall et al., 2004). *LAC1* has a unique C-terminal motif that localizes the protein to the cell wall of *C. neoformans* at physiological pH (7.4; Waterman et al., 2007). *LAC2* is truncated in the C-terminal region and is located in the cytosol under normal conditions but can locate to the cell wall in the absence of *LAC1* (Missall et al., 2004). Both *LAC1* and *LAC2* genes contribute to melanization.

Here, we found that *APP1, LAC1*, and *LAC2* genes are all upregulated old *C. neoformans* cells (10 generations old) compared to young cells (0–2 generations old). Interestingly, all three mutants exhibited shorter median lifespans. Furthermore, our data demonstrates that when knockout mutant strains were aged to 10 generations, they no longer exhibited enhanced age-dependent resistance to killing by antifungals, macrophages, or *Galleria mellonella* worms. Furthermore, we also found that *LAC2* is not just a redundant gene to compensate for *LAC1*, but rather, it contributes to age-dependent resilience distinct from *LAC1*.

## Materials and Methods

### Strains and Media

Strains and sources of strains used in this study are shown in Table 1. All strains were stored in 30% glycerol at −80°C. When needed, strains were struck on Yeast-Peptone-Dextrose (YPD) agar plates (BD), grown at 37°C and stored at 4°C. Media used in this study is listed in Table 2. All liquid cultures were grown at 37°C, shaking at 150 rpm.

**TABLE 1:** Strains used in this study.

**Strain**	**Source**
H99	Dr. John Perfect (Duke University)
Kn99α	Dr. Maurizio Del Poeta (Stony Brook University)
H99Δa*pp1*	Dr. Maurizio Del Poeta (Stony Brook University)
H99Δ*lac1*	Dr. Peter Williamson (National Institutes of Health)
Kn99αΔ*lac2*	Dr. Maurizio Del Poeta (Stony Brook University)

**TABLE 2:** Media used in this study.

**Media**	**Components per 1 Liter or source**
Yeast-Peptone-Dextrose (YPD)	BD – Unmodified
Sabaroud Dextrose (SAB)	BD – Unmodified
Melanization Media (MM)	4 g KH_2_PO_4_, 2.5 g MgSO_4_⋅7H_2_O, 0.975 g Glycine, 3 g Dextrose, 1 mg Thiamine, 1 mM L-DOPA
Synthetic Media (SM)	1.7 g yeast nitrogen base without amino acids, 1 g drop out mix, 0.4% ethanol, 5 g (NH_4_)_2_SO_4_, 3.3 g NaCl, 20 g glucose
RPMI 1640	Gibco – unmodified
DMEM	Gibco – modified with 10% heat inactivated Fetal Bovine Serum, 10% NCTC (Gibco), 1% non-essential amino-acids, and 1% Penicillin-Streptomycin

### Replicative Lifespan and Cell Size Measurement

The replicative lifespan (RLS) of all strains was determined by traditional microdissection methodology (Bouklas et al., 2013). Briefly, 25–30 cells were lined up on a YPD agar plate using a 25 μm fiber-optic needle (Cora Styles) on a tetrad dissection Axioscope A1 microscope (Zeiss) and grown at 37°C. After 1 division, naïve cells were separated from their mothers and designated as starting mother cells. These cells were then tracked and after every division (every 60–120 min), naïve daughter cells were counted and removed to allow the mothers to continue to divide. In between divisions, cells were incubated at either 37°C or 4°C overnight to slow growth and prevent excessive replication. As cells reached desired generational age, images were taken of the cells on top of an EVOS FL Auto microscope (Thermo Fisher Scientific) and cell sizes were measured using FIJI opensource software.

### Isolation of Old Cells

Previously described methods for isolation of old and young cells were slightly modified (Bouklas et al., 2013). Briefly, exponential cells were washed and labeled with 8 mg/mL Sulfo-NHS-LC-LC-Biotin (Thermo Scientific) for 1 h at room temperature. After 1 h, cells were washed with PBS and labeled with 20 μL anti-biotin (Miltenyi Biotec) per 10^7^ cells for 1 h at 4^°^C. After labeling, cells were grown in either sabaroud dextrose (SAB) (Difco) media, melanization media (MM), or synthetic media (SM) as outlined to a specific generation. Recovery of generationally aged mother cells was done using LS magnetic columns (Miltenyi Biotec). The negative fraction that washed off the column was used as the young control group as they were exposed to the same manipulation as old cells retained in the magnetic column.

### Quantitative Reverse Transcriptase Polymerase Chain Reaction (qPCR)

RNA was isolated from both young (0–3 generation) and old (10 generation) cells grown in SAB using Qiagen RNAeasy Kit following manufacturer’s guidelines. Nanodrop (Biospectrophotometer, Eppendorf) was used to quantify the isolated RNA. An absorption ratio (A260/A280) of 2.0 was considered pure and good quality RNA. 250 ng of RNA was converted to cDNA using Verso cDNA Kit (Thermoscientific) following manufacturer’s guidelines. Oligo-dT was used as the primer for the preparation of cDNA. After this, cDNA was diluted 1:5 with nuclease free water (Hyclone) and subjected to qPCR (Roche) utilizing Power SYBR Green Master Mix (Applied Biosystems) following manufacturer’s guidelines. The oligonucleotides used for this assay are listed below. Housekeeping gene *ACT1* was used as an internal control. Data was normalized to the gene expression in the young (0–3 generation) cells of both wildtype strains. Data was analyzed using ΔΔCt method as previously described (Livak and Schmittgen, 2001). The following primers were used:

*ACT1:* F5′-CCCACACTGTCCCCATTTAC-3′, R5′-AACCAC GCTCCATGAGAATC-3′; *APP1:* F5′-CCAAACTGCGTTACTC AGCA-3′, R5′-TAATGCTGCTTTCCCCATTC-3′; *LAC1*: F5′*-*TT TGGGTCCGCCCCTTAATTATC-3′, R5′-GGATAGGTGCATG AGGAGGA-3′; *LAC2*: F5′-TATCCTCCTCCCGAGAT-3′, R5′-GCATCCCCTTCTTTTCCTTC-3′.

### Macrophage Phagocytosis and Killing

J774.16 macrophages were cultured in DMEM (Gibco) media containing 10% heat inactivated Fetal Bovine Serum (FBS), 10% NCTC (Gibco), 1% non-essential amino-acids and 1% Penicillin-Streptomycin. 5 × 10^5^ macrophages/well were plated in 96-well flat-bottomed plate (Costar) and incubated for 24 h at 37^°^C with 10% CO_2_. After 24 h, macrophages were activated with LPS and IFNγ as described previously (Jain et al., 2009). After activation, the macrophages were washed three times and fresh DMEM media was added. In separate tubes, 10^5^ young (0–3 generations) or old (10 generation) cells from each *Cryptococcus* strain grown in SAB were incubated with either 10% normal human serum (NHS) or 18B7 antibody for 5 min for opsonization. Opsonized cells were added directly onto the macrophages at a MOI of 1:1 and incubated for 1 h at 37^°^C with 10% CO_2_. After 1 h of phagocytosis, all wells were washed three times with PBS. After 1 h of phagocytosis and washing, half the wells of macrophages were lysed with sterile water and *C. neoformans* cells were plated on YPD agar plates to determine the number of colony-forming units (CFUs) engulfed at time 0. In the second half of wells, fresh DMEM was added and cells were allowed to kill any engulfed cells for 1 h at 37^°^C with 10% CO_2_. After 1 h of killing, macrophages were lysed with sterile water, and surviving *C. neoformans* cells were plated on YPD agar plates to determine the number of CFUs after phagocytic killing. All YPD plates were incubated for 48 h at 37^°^C. CFUs were counted and percent killing was calculated as #⁢CFU⁢post⁢phagocytosis⁢time⁢ 0-#⁢CFU⁢time⁢ 1⁢h#⁢CFU⁢post⁢phsgocytosis⁢time⁢ 0×100%.

For phagocytic index, Giemsa staining was used to identify the number of *C. neoformans* cells in each macrophage. Images of cells were taken with EVOS FL Auto microscope (Thermo Fisher Scientific). Phagocytic index was calculated as $\frac{\#\,\mathrm{Cn}\,\mathrm{engulfed}}{\mathrm{Total}\,\mathrm{number}\,\mathrm{of}\,\mathrm{Macrophages}}\times 100\,\mathrm{the}\,\mathrm{number}\,\mathrm{of}\,C.n\,e\,o\,f\,o\,r\,m\,a\,n\,s$ cells engulfed by the macrophages divided by the total number *of macrophages engulfing the C. neoformans* cells multiplied by 100 (Zaragoza et al., 2003).

### *Galleria mellonella* Infection

*Galleria mellonella* infection was carried out as previously described (Bouklas et al., 2015). *C. neoformans* cells grown in SAB were washed and diluted in PBS to 10^6^ cells/mL. 10 μL of the cell suspension was injected into each *G. mellonella* worm (Vanderhorst Wholesale Inc., St. Mary’s, OH, United States) and 20 worms were used for each strain. One set of 20 worms were injected with PBS as negative control and another set of twenty larvae were neither injected with PBS nor with *Cryptococcal* cells. This group was used as a quality control for the worms. Survival was noted for a week.

### Melanization

To assess melanization, cells were grown in melanization media (MM) for the specified amount of time. Pigmentation was measured by spinning cells down into a PCR tube and capturing an image of the pellets. Pellet pigmentation was analyzed by histogram analysis on a scale of 0-255 (0 = true black, 255 = true white) using FIJI opensource software (Schindelin et al., 2012). Melanization between strains was normalized by ensuring the starting culture concentrations and time in MM was the same across all strains.

### Antifungal Killing

Antifungal Killing assays were done as previously described (Jain et al., 2009). Briefly, cells were either melanized and grown in MM or grown in SM. Cells were grown to 10 generations and isolated as described above. The unlabeled fraction served as the young control (0–3 generation) against the 10 generation old cells. Young and old, and melanized and unmelanized cells of all strains were subjected plated in 96 well plates at 10^4^ cells/mL and were subjected to 0.5 μg/mL of amphotericin B for 3 h. After 3 h, cells were diluted and plated on YPD plates. After plates were incubated for 48 h at 37°C, the number of colony forming units (CFUs) were counted and compared to CFUs of plates incubated with no antifungals ($\%\mathrm{killing}=\frac{\#\,\mathrm{CFU}\,\mathrm{with}\,\mathrm{drug}}{\#\,\mathrm{CFU}\,\mathrm{without}\,\mathrm{drug}}$).

### Statistics

Statistical analysis was performed using Graph Pad Prism 6.0 and 8.0. The names of the statistical tests performed for each experiment are listed in the figure legends.

## Results

### Upregulation of Genes With Age

**FIGURE 1:**
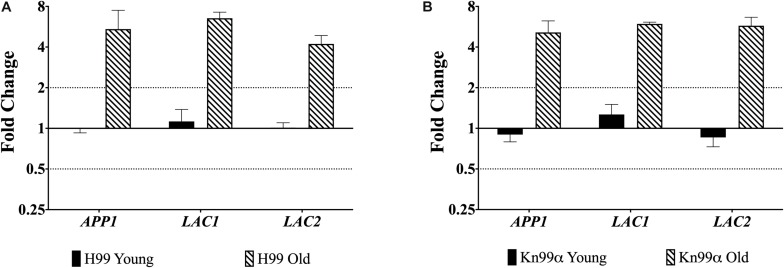
Increased expression of virulence genes in old cells. Expression of genes *APP1*, *LAC1*, and *LAC2* in young (0–3 generations) and old (10 generations) cells from both wildtype H99 **(A)** and KN99α **(B)** was analyzed using qPCR. Data was normalized to the gene expression in young cells. Housekeeping gene *ACT1* was used as an internal control. Twofold up or twofold downregulation, marked by the dotted lines, was considered a significant fold change difference. Error bars signify standard deviation of technical replicates.

First, we used qPCR to analyze the expression of genes *APP1*, *LAC1*, and *LAC2* in the young (0–3 generations) and old (10 generation) cells from both wildtype strains H99 and KN99α. All three genes were significantly (>two-fold) upregulated in old wildtype cells when compared to young cells (Figure 1).

### Macrophage Phagocytosis

**FIGURE 2:**
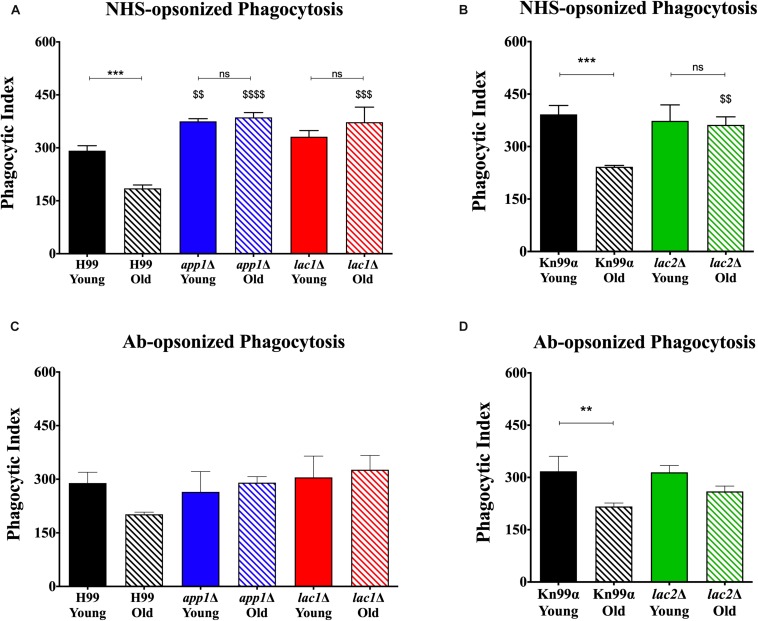
Decreased complement- and antibody-mediated macrophage phagocytosis of old wildtype cells compared to young and mutant cells. Phagocytic index was measured as the number of *C. neoformans* cells engulfed by macrophages divided by the total number of macrophages multiplied by 100. The assays were performed by opsonization of the fungal cells with 10% normal human serum **(A,B)** or by 18B7 antibody **(C,D)**. The assays were done in biological triplicate and error bars signify standard deviation between replicates. *^∗∗^p* < 0.01, *^∗∗∗^p* < 0.001 by one-way ANOVA. Bars without any ^∗^ are non-significant. ^$$^*p* < 0.01, ^$$$^*p* < 0.001, ^$$$$^*p* < 0.0001 by one-way ANOVA when compared to wildtype counterpart.

Macrophage-mediated phagocytosis assays were performed by opsonizing the fungal cells with either 10% normal human serum (NHS; Figures 2A,B) or by opsonizing the fungal cells with 18B7 antibody (Ab; Figures 2C,D; Casadevall et al., 1998). As expected, under NHS-mediated opsonization conditions, old wildtype cells were phagocytosed significantly less than young wildtype cells (H99: 185 vs. 292%, respectively; KN99α: 242 vs. 392%, respectively). This age-related resistance to phagocytosis is not observed with old cells isolated from the mutants *app1*Δ, *lac1*Δ, and *lac2*Δ. As expected, young *app1*Δ cells exhibited significantly higher phagocytosis compared to wildtype (375 vs. 292%, respectively). In contrast, neither young *lac1*Δ (331%) nor young *lac2*Δ (373%) cells exhibited altered phagocytosis compared to wildtype (H99: 292%; KN99α: 392%). In contrast, old cells of all three mutants were phagocytized significantly more compared to the old cells of the respective wild type (*app1*Δ: 386 vs. 185%; *lac1*Δ: 373 vs. 185%; *lac2*Δ: 362 vs. 242%).

Next, antibody-mediated opsonization was compared. These experiments indicated a significant decrease in phagocytosis of old KN99α cells when compared to young cells (217 vs. 318%) and the same trend of impaired phagocytosis of old H99 cells was indicated (202 vs. 289%). Again, analogous to the NHS-mediated opsonization, we did not observe significant differences in phagocytosis between the young and old *app1*Δ, *lac1*Δ, and *lac2*Δ mutant cells. Unlike NHS-mediated opsonization, no significant differences were observed in phagocytosis of old wildtype and old mutant cells when opsonized with mAb 18B7.

### Macrophage-Mediated Killing

**FIGURE 3:**
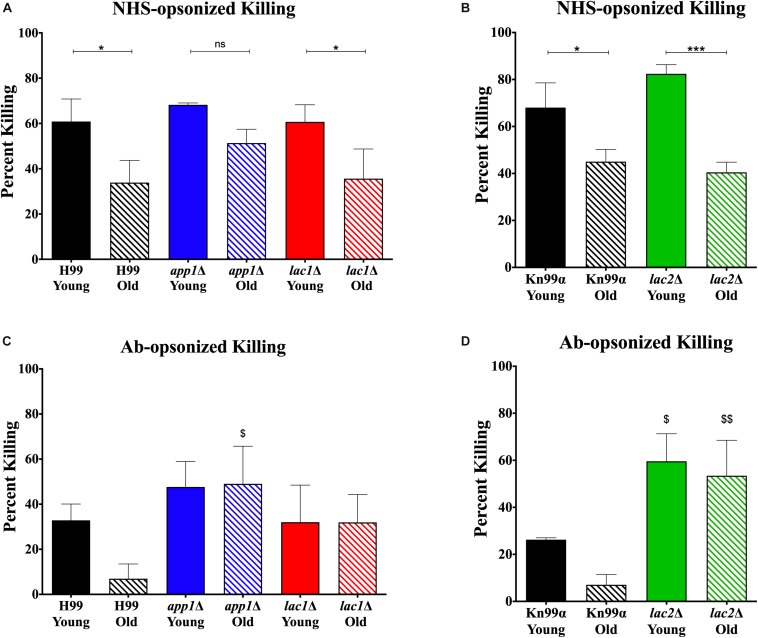
Altered complement-mediated macrophage killing of old cells and mutant cells. Phagocytized cells were incubated for 1 h in macrophages to allow for phagocytic killing. Surviving *C. neoformans* cells were plated on YPD agar plates and CFUs were counted after 48 h incubation at 37^°^C and normalized to CFUs at time 0 before 1 h of killing. *C. neoformans* cells were opsonized with 10% normal human serum **(A,B)** or by 18B7 antibody **(C,D)**. Assays were done in biological triplicate and error bars signify standard deviation between replicates. One-way ANOVA was performed to analyze the statistical significance. ^∗^*p* < 0.05, *^∗∗∗^p* < 0.001 by one-way ANOVA. ^$^*p* < 0.05, ^$$^*p <* 0.01 by one-way ANOVA compared to wildtype counterpart.

Since altered phagocytosis can but does not necessarily affect macrophage-mediated killing, both young and old cells isolated from wildtype and mutant strains were subjected to 1-h killing by macrophages and quantification was corrected for the altered phagocytosis index. As expected, a decrease in macrophage-mediated killing was observed for NHS-opsonized old cells compared to young cells of both H99 (35 vs. 65%, Figure 3A) and KN99α (40 vs. 65%, Figure 3B). Similar to wildtype, NHS-opsonized, old *lac1*Δ and *lac2*Δ mutant cells also showed a significant decrease in macrophage-mediated killing (*lac1*Δ: 36 vs. 61%; *lac2*Δ: 40 vs. 82%). However, no significant change in macrophage-mediated killing was observed between old and young *app1*Δ cells (51 vs. 68%).

In contrast, antibody opsonization did not result in a significant difference in killing between young and old cells for any strain (Figures 3C,D) suggesting that serum mediated uptake may play a more important role in the observed age-related loss of resistance in mutants.

### Virulence in *Galleria mellonella*

**FIGURE 4:**
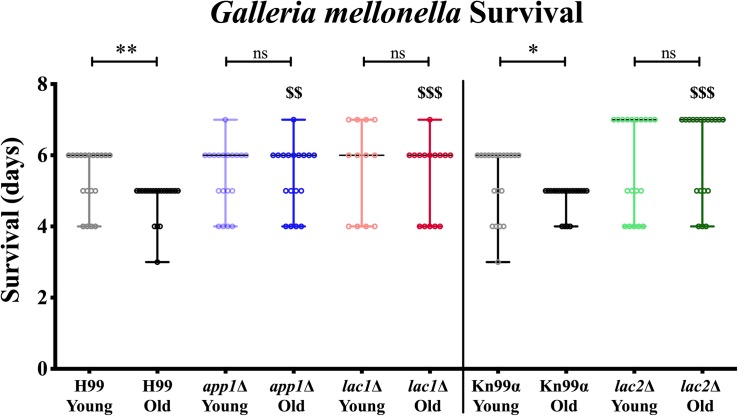
Young cells and mutant strains are less virulent in *Galleria mellonella*. 10^6/^ml cells were injected each in 20 *Galleria* larvae and survival was noted for 7 days. ^∗^*p* < 0.05, *^∗∗^p* < 0.01 by log-rank test. ^$$^*p <* 0.01, ^$$$^*p* < 0.001 by log-rank test compared to wildtype counterpart.

Next, we studied the effects of aging in these mutants in the *Galleria* infection model. Each larva was infected with 10^4^ cells as outlined in the materials and methods. Larvae infected with old wildtype cells survived less days than the larvae infected with young wildtype cells (5 vs. 6 days, Figure 4, and Table 3). No significant change in survival was observed in the larvae infected with young vs. old mutant cells. Similarly, no significant differences were observed in the survival of the larvae infected with young wildtype cells vs. young mutant cells. Consistent with the loss of impaired phagocytic uptake and killing of mutants, we observed decreased virulence of old mutant cells compared to their respective wildtype aged cells. Specifically, significant differences were observed in survival of the larvae infected with old H99 vs. old *app1*Δ cells (5 vs. 6 days) and old H99 vs. old *lac1*Δ cells (5 vs. 6 days). Similarly, a significant difference was observed in survival of larvae infected with the old KN99α cells vs. old *lac2*Δ cells (5 vs. 7 days).

**TABLE 3:** Young cells and mutant strains are less virulent in *Galleria mellonella*.

**Strain name**	**Age**	**Galleria survival (Median) days**	***P*-value**	**Significance**	**Remarks**
H99	Y	6	0.0015	S	Aging significantly increased virulence
				
	O	5			

*lac1*Δ	Y	6	0.55	NS	Age-associated increased virulence disappeared
				
	O	6			

*app1*Δ	Y	6	0.55	NS	App1 important in age-dependent resilience
				
	O	6			

H99	Y	6	0.0943	NS	Lac2 compensates for Lac1 in young cells
	
*lac1*Δ	Y	6			

H99	Y	6	0.5238	NS	App1 works via complement and *Galleria* lacks full complement system
	
*app1*Δ	Y	6			

H99	O	5	0.0002	S	Lac2 failed to compensate for loss of Lac1 in old age
	
*lac1*Δ	O	6			

H99	O	5	0.0022	S	App1 needed for age-dependent virulence
	
*app1*Δ	O	6			

KN99α	Y	6	0.004	NS	Aging significantly increased virulence
				
	O	5			

*lac2*Δ	Y	7	0.47	NS	Lac2 important in age-dependent resilience
				
	O	7			

KN99α	Y	6	0.02	NS	Lac1 compensates for Lac2 in young cells
	
*lac2*Δ	Y	7			

KN99α	O	5	0.0004	S	Lac1 failed to compensate for loss of lac2 in old age
	
*lac2*Δ	O	7			

### Melanization

**FIGURE 5:**
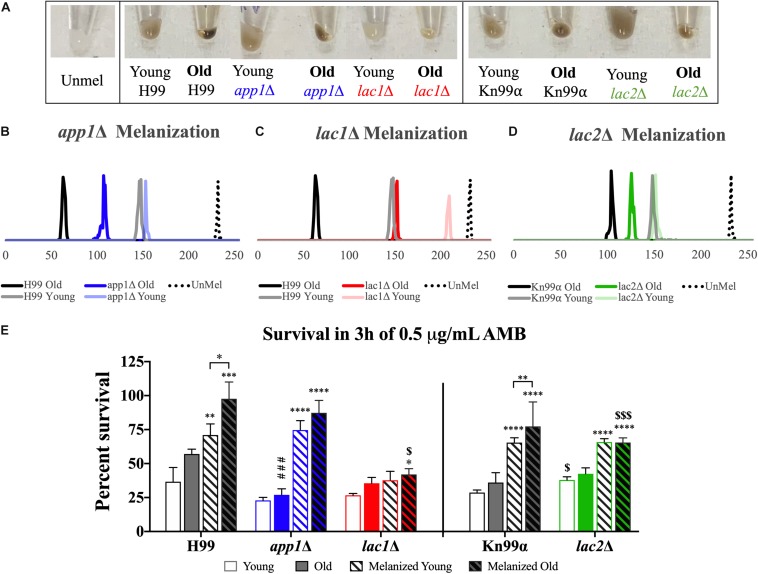
Melanization increases with age which enhances antifungal resistance. Wildtype and mutant cells were aged to 10 generations in melanization media. Old cells were separated from the young, negative fraction and melanization was compared by imaging cell pellets **(A)** and quantifying darkness using black to white histogram analysis (on a scale from 0 to 255. 0, true black, 255, true white). Melanization of young and old fractions of *app1*Δ **(B)**, *lac1*Δ **(C)**, and *lac2*Δ **(D)** were compared to melanization of young and old fractions of their respective wildtype strains. Fractions of young and old cells from each strain grown in control and melanization media were subjected to 3 h of killing by 0.5 μg/mL AMB **(E)**. Percent survival was calculated for each population which was then normalized to the young wildtype strain grown in control media. ^∗^*p* < 0.05, ^∗∗^*p* < 0.01, ^∗∗∗^*p* < 0.001, ^∗∗∗∗^*p <* 0.0001 by one-way ANOVA compared to young cells of the same strain grown in control media. ^$^*p* < 0.05, ^$$$^*p* < 0.001 by one-way ANOVA compared to wildtype counterpart.

In order to quantify melanization, cell pellets were assessed using histogram analysis to determine intensity values on a black to white scale. On such a black to white intensity scale, true black has an intensity value (IV) of 0 and true white has an IV of 255. When strains were aged in melanization media, old cells melanized to a higher degree than the young, negative fraction (Figures 5A–D). Young *app1Δ* (IV = 152) and *lac2*Δ (IV = 150) cells melanized to the same degree as young wildtype cells (IV = 147 for both H99 and Kn99α) whereas young *lac1*Δ cells (IV = 208) were closer to unmelanized cells (IV = 231). H99 old (62) and young (147) cells showed a shift of 85 in magnitude and Kn99α old (103) and young (147) cells showed a shift of 44 in magnitude. None of the mutant strains had as large of a difference in IV as their respective wildtype strains. Compared to H99, *app1*Δ young (152) and old (106) cells only showed a shift of 46 in magnitude and *lac1*Δ young (208) and old (150) cells showed a shift of 58 in magnitude. *lac2*Δ young (150) and old (125) cells showed the smallest shift of all strains with only a shift of 25 in magnitude.

### Antifungal Killing

Since melanization increases resistance to amphotericin B (AMB), we assessed if melanization in old cells further enhances this resistance. Yeast cells were subjected to 3 h of killing by 0.5 μg/mL of AMB, a concentration 4-fold higher than minimum inhibitory concentration (MIC) for all strains (MIC = 0.0625 μg/mL, data not shown). These data confirm findings of previous studies (van Duin et al., 2002) that melanization augments resistance to AMB in wildtype young cells (H99 unmelanized = 36.64%, melanized = 71.01% survival; Kn99α unmelanized = 28.69%, melanized = 65.44% survival). Importantly, melanization markedly increases the enhanced resistance of older cells relative to young melanized cells (H99 Y = 71.01%, O = 97.65% survival; Kn99α Y = 65.44%, O = 77.39% survival) (Figure 5E).

Old *app1*Δ cells, exhibited significantly less resistance to AMB than old wildtype cells (26.96 vs. 57.04% survival, respectively). Melanization, however, significantly enhanced antifungal resistance of young and old *app1*Δ mutant cells. Interestingly there was no significant difference between young and old melanized *app1*Δ cells (74.65 vs. 87.36%, respectively).

As expected *lac1*Δ mutant cells exhibited minimal melanization. Hence, no difference between unmelanized young and melanized young *lac1*Δ cells was noted. The minimally melanized old *lac1*Δ cells were therefore significantly more susceptible than melanized old wildtype cells (42.01 vs. 97.65% survival, respectively).

Similar to *lac1*Δ cells, there was no significant difference between young and old unmelanized *lac2*Δ cells or between young and old melanized *lac2*Δ cells. Melanization of *lac2*Δ cells did, however, increase the resistance of young cells (65.82 vs. 37.92% survival, respectively) and old cells (65.46 vs. 42.50% survival, respectively). Lastly, also similar to *lac1*Δ cells, old melanized *lac2*Δ cells were more susceptible than old melanized wildtype cells (65.46 vs. 77.39% survival, respectively).

### Replicative Lifespan and Cell Size

**FIGURE 6:**
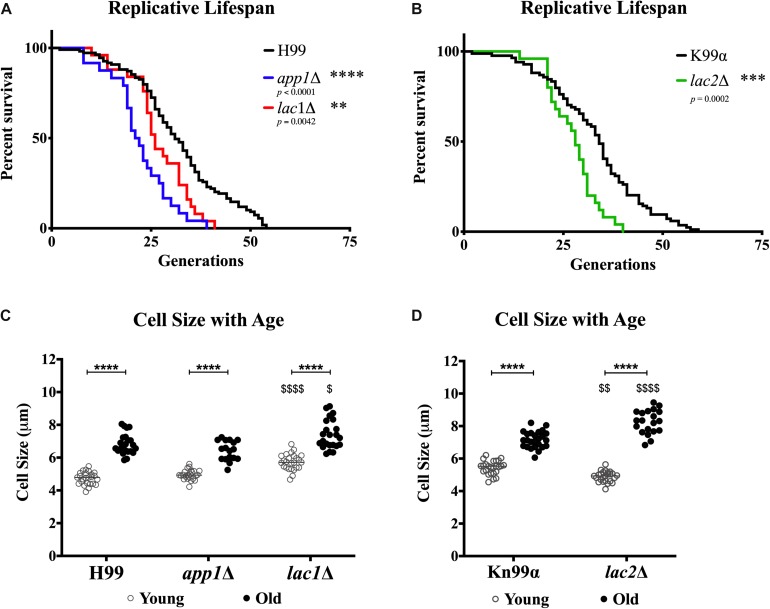
Decreased lifespan of mutants and increased cell size with age. Replicative lifespans were determined for each mutant strain and compared to its respective wildtype **(A,B)**. Cell sizes were measured in the young (0–3 generations) and old (10 generations) fractions of cells after separation to assess the cell size of each population **(C,D)**. ^∗∗^*p* < 0.01, ^∗∗∗^*p* < 0.001, ^∗∗∗∗^*p* < 0.0001 by one-way ANOVA. ^$^*p* < 0.05, ^$$^*p* < 0.01, ^$$$$^*p* < 0.0001 by one-way ANOVA compared to wildtype counterpart.

In order to determine whether the changes in susceptibility seen with age were associated with changes in lifespans or cell size, we determined the replicative lifespan (RLS) (Figure 6A) and assessed the size of mutants and wildtype cells (Figure 6B). All mutant strains exhibited shortened median RLS compared to wildtype (Figure 6C). This was most pronounced for *app1*Δ cells, which had a median RLS of 21.5 generations (33.9% loss). Both *lac1*Δ cells and *lac2*Δ cells also exhibited shorter lifespans (16.1% loss, and 17.6% loss, respectively).

In all strains a significant increase in cell size was observed with advanced generational age (H99 Y = 4.736 μm, O = 6.824 μm; *app1*Δ Y = 4.952 μm, O = 6.424 μm; *lac1*Δ Y = 5.732 μm, O = 7.399 μm; Kn99α Y = 5.444 μm, O = 7.162 μm; *lac2*Δ Y = 4.894 μm, O = 8.259 μm) (Figure 6D). Furthermore, *lac1*Δ cells were larger at young and old age compared to wild-type and *lac2*Δ cells were also significantly larger at old age compared to wildtype. Interestingly, no significant difference between young wildtype and *app1*Δ cells, or old wildtype and *app1*Δ cells was noted despite the significantly shortened lifespan of *app1*Δ cells. Similarly, young *lac2*Δ cells were significantly smaller than wildtype despite the significantly shortened lifespan.

### Gene Regulation With Age in Mutants

**FIGURE 7:**
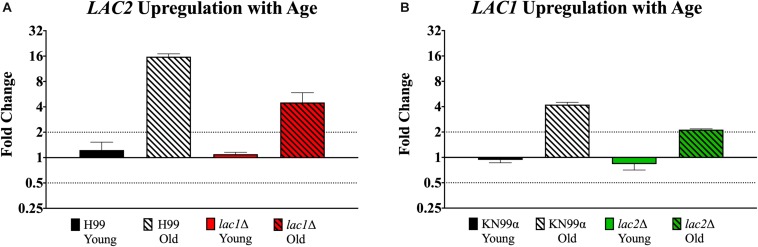
Increased expression of *LAC1* and *LAC2* genes in old cells. Expression of *LAC2* in young (0–3 generations) and old (10 generations) wildtype H99 and *lac1*Δ cells **(A)**. Expression of *LAC1* in young (0–3 generations) and old (10 generations) wildtype KN99α and *lac2*Δ cells **(B)**. Data was normalized to the gene expression in young cells. Housekeeping gene *ACT1* was used as an internal control. Twofold up or twofold downregulation, marked by the dotted lines, was considered a significant fold change difference. Error bars signify standard deviation of technical replicates.

qPCR was used to evaluate potential compensation between laccase genes in the respective mutant. Though old *lac1*Δ mutant cells showed increased expression of *LAC2* compared to young *lac1*Δ cells (4.51-fold vs. 1.09-fold, Figure 7A), the expression of *LAC2* in old *lac1*Δ mutant cells was much lower than the expression of *LAC2* in old wildtype cells (4.51-old vs. 15.75-fold, Figure 7A). Similarly, old *lac2*Δ cells showed increased expression of *LAC1* when compared to young cells (2.13-fold vs. 0.83-fold, Figure 7B) and the expression of *LAC1* in old *lac2*Δ cells was much lower than *LAC1* expression in old wildtype cells (2.13-fold vs. 4.23-fold, Figure 7B). Importantly, these results suggest that *LAC2* expression in old *lac1*Δ cells is higher than *LAC1* expression in *lac2*Δ cells.

## Discussion

Previous studies have demonstrated that generationally older *C. neoformans* accumulate during infection, and are more virulent, and more resistant to antifungals (Bouklas et al., 2013, 2017b). This may result from a thickened cell wall, increased cell size, or upregulation of drug exporters in generationally old cells. In this study, we analyzed the roles of three cell-wall-associated proteins, App1, Lac1, and Lac2, in age-dependent virulence and antifungal tolerance in *C. neoformans*. Their transcription was significantly upregulated in 10-generation old cells, suggesting the importance of these proteins in generationally old cells.

*APP1* encodes for an antiphagocytic protein and provides protection to the fungal cells against macrophage phagocytosis. As expected, the phagocytic index of young *app1*Δ cells was significantly lower compared to the phagocytic index of young wildtype cells. This confirms previous results which identify *APP1* as an important player in preventing phagocytosis (Luberto et al., 2003). The phagocytic index for 10-generation-old, NHS-opsonized wildtype cells was significantly lower compared to young wild type cells. However, old *app1*Δ cells exhibited no significant difference in the phagocytic index when compared to that of young *app1*Δ cells. The phagocytic index for 10-generation-old, Ab-opsonized wildtype cells was also lower compared to young wild type cells. However, there was no significant difference in the phagocytic index between *app1*Δ and wildtype cells. This difference between complement- and antibody-mediated phagocytosis is likely due to the fact that App1 inhibits phagocytosis by a complement-mediated and not an Ab-mediated mechanism.

App1 is similarly important in preventing macrophage-mediated killing of generationally older cells. Old wildtype cells are killed significantly less than young cells, however, we found no significant difference in macrophage-mediated killing of young and old *app1*Δ cells. This is true for both Ab-opsonized and NHS-opsonized cells. Thus up-regulation of *APP1* in generationally older *C. neoformans* cells contributes to both the decreased phagocytosis and decreased macrophage-mediated killing in generationally older cells *in vitro*.

We found further support for these findings *in vivo* in the *G. mellonella* insect model. Although, *Galleria* do not have a true complement system they utilize complement-like proteins (Tsai et al., 2016). Survival of *Galleria* worms infected with *app1*Δ cells was not altered by aging whereas older wildtype cells killed the worms faster. This finding is consistent with the *in vitro* killing assays and further indicates that App1 is an important virulence factor that contributes to enhanced resilience of older generation of cells.

In addition to the role of App1 in inhibiting phagocytosis and macrophage-mediated killing, Qureshi et al. (2012) hypothesized that App1 may also play a role in melanization as it has amyloid properties and amyloids have been shown to play a role in melanin biosynthesis in other species. Interestingly, we found that young *app1*Δ cells melanized to the same extent as wildtype cells, whereas old *app1*Δ cells exhibited lower levels of melanization compared to old wildtype cells. Regardless, melanization of both young and old *app1*Δ cells increased their resistance to AMB killing.

Of interest, App1 seems to play a role in age-dependent resistance to AMB as old unmelanized *app1*Δ cells do not survive significantly more than young unmelanized cells and in fact, the old *app1*Δ cells survive significantly less than old wildtype cells. Furthermore, though melanization enhances *app1*Δ cells resistance against AMB killing, there is still no significant difference between young and old melanized *app1*Δ cells. Future studies are required to determine whether loss of App1 alters cell wall structure as App1 is located in the cell wall (Qureshi et al., 2012). If true, it would be interesting to further explore if such changes in the cell wall’s composition may also have an effect on the cell membrane, which is below the cell wall. Others have described that changes in cell membrane affect the cell wall and alter its sensitivity to caspofungin and congo red, which target the cell wall (Mesa-Arango et al., 2016; Bhattacharya et al., 2018).

In addition to *APP1*, we studied the effects of the laccase enzyme encoding genes *LAC1* and *LAC2* on age-dependent resilience. Lac1 typically localizes in the cell wall but it is highly pH dependent. In *C. neoformans* cells located in the brain (physiological pH around 7.2-7.4), Lac1 is almost exclusively found in the cell wall. At lower pHs, however, such as pH of 5 as seen in macrophages, Lac1 tends to get trapped in cytoplasmic vesicles (Waterman et al., 2007). Interestingly, it has been shown that Lac1 plays an important role in extrapulmonary dissemination to the brain but it does not contribute to pulmonary survival or persistence (Noverr et al., 2004). Lac2 is a protein similar to Lac1 and both of them play an important role during melanization. For this study, we obtained the mutant strains lacking *LAC1* and *LAC2.*

First, we assessed whether *LAC1* or *LAC2* are important in macrophage phagocytosis. No significant difference in phagocytosis between young wildtype and mutant cells was documented. Old *lac1*Δ and *lac2*Δ cells, however, were phagocytized significantly more than old wildtype cells indicating both *LAC1* and *LAC2* are important in the age-dependent resilience to macrophage phagocytosis. This was observed for NHS-opsonized cells but not Ab-opsonized cells, suggesting that age-dependent resilience to phagocytosis mediated by *LAC1* and *LAC2* may be through a complement-mediated mechanism.

Because *LAC1* and *LAC2* play a role in phagocytosis, we then assessed whether *LAC1* and *LAC2* were important for macrophage-mediated killing. Both wildtype and mutant populations were more resistant to macrophage killing when aged compared to respective young populations. When cells were opsonized with antibody, however, old *lac1*Δ lost their age-dependent resilience as they were killed at the same rate as young *lac1*Δ cells. Furthermore, both young and old *lac2*Δ were killed at higher rates compared to their respective wildtype populations and old *lac2*Δ cells lost their age-dependent resilience to killing compared to their young counterparts. Taken together, both *LAC1* and *LAC2* seem to play a role in age-dependent resilience to phagocytic killing but only when opsonized by antibody and not complement. Furthermore, *LAC2* may play an important role in resistance against antibody-mediated phagocytic killing.

To further analyze the virulence of the *lac1*Δ and *lac2*Δ mutants *in vivo*, young and old mutant cells were injected into *Galleria* and survival was assessed. Overall, we observed no significant changes in virulence between young mutants and young wildtype cells. This suggests, in conjunction with our phagocytosis results, that in young cells, Lac1 and Lac2 may be compensating for each other when one gene is absent. However, significant changes in age-dependent virulence were observed. Both *lac1*Δ and *lac2*Δ mutant populations showed a loss of age-dependent virulence as both young and old populations of either mutant strain killed *Galleria* at the same rate. Furthermore, old *lac1*Δ and *lac2*Δ cells were significantly less virulent than wildtype old cells, further indicating Lac1 and Lac2 are important in mediating age-dependent resilience.

Since the most important role of Lac1 and Lac2 is melanization, we analyzed melanization in young and old *lac1*Δ and *lac2*Δ cells and compared them with their respective wildtypes. As expected, loss of either *LAC1* or *LAC2* reduced the ability of cells to melanize. Previously it was thought that *LAC1* was the main contributor and *LAC2* served a less important, redundant role (Missall et al., 2004). This is likely because previous studies only focused on young cultures. Looking at generationally older cells, we see *LAC2* plays an important role in melanization at older ages. *lac1*Δ mutant cells do not melanize well overall, but old cells are able to melanize to a greater extent (shift of 58 IV units between young and old) compared to *lac2*Δ mutant cells (shift of 25 IV units between young and old). Furthermore, when young and old melanized cells were subjected to AMB for 3 h, old *lac1*Δ mutant cells were 11.4% more resistant to killing compared to young. There was no difference, however, in resistance of old and young *lac2*Δ mutant cells (65.5 vs. 65.58%, respectively). Taken together, *LAC2* may partially compensate for loss of *LAC1* in old age. The loss of resistance to AMB in old laccase mutant cells may again be explained by altered composition to the cell wall as seen in the *app1*Δ mutant cells. However, Lac2 is not typically found in the cell wall unless Lac1 is missing so altered cell wall composition in the mutants cannot be the only factor contributing to this loss of AMB resistance in old age.

Since we observed that each of these genes play a significant role in age-dependent resistance, we asked the question whether App1, Lac1, or Lac2 play any role in altering the replicative life span of *C. neoformans.* In *C. neoformans*, strains can only undergo a finite number of divisions before senescence, the cumulative total of which is termed the replicative lifespan (RLS) (Bouklas et al., 2013). When the RLS of a strain is shifted in either direction, the relative age of a 10-generation old cell shifts and the respective age-dependent resilience is altered (Bouklas et al., 2017b). When a wildtype strain lives to be on average 30 generations, 10 generations is one third it’s lifespan. If that strain is mutated and its average RLS shifts to only 20 generations, a 10-generation old cell is now through half of its lifespan making it relative older. We have shown previously that this decrease in RLS also alters the strains age-dependent resilience as 10 generation cells from the mutant with a shortened RLS are more resistant to macrophage killing than 10-generation wildtype cells (Bouklas et al., 2017b). To ensure the loss in age-dependent resistance of our mutant strains was not solely due to a shift in RLS, we determined RLS of each mutant. All three mutants exhibited decreased median RLSs compared to their respective wildtypes. Since 10-generation cells of all mutants are relatively older than their wildtype counterparts, their age-dependent resilience should be higher than the wildtype. Similarly, 10-generation old mutant cells are as large or significantly larger than 10-generation wildtype cells. Taken together, we conclude the decrease and loss in age-dependent resilience of mutant strains is likely not due to the change in RLS or their cell size.

Though it is difficult to hypothesize why these virulence factors affect RLS, it has been found that *APP1* transcription is controlled by diacylglycerol (DAG) through the transcription factor *ATF2* (Mare et al., 2005). In *C. neoformans*, DAG also activates Pkc1 (Heung et al., 2004), and Pkc1 regulates Laccase (Heung et al., 2005) and Sir2 silencing (Lee et al., 2013) (an age-regulating gene). Thus, increased DAG would increase activation of App1 through Atf2, as well as Laccase activity and Sir2 silencing through Pkc1, which could result in more App1 being secreted, increased melanization, and longer replicative lifespan. Future studies to investigate these potential mechanisms are planned to discern the common pathway(s) that regulate these genes and their transcription factors to better understand how they contribute to age-dependent resilience.

In conclusion, App1, Lac1, and Lac2 each play a significant role in age-dependent resilience and regulation of replicative life span. App1 is more important for age-dependent resistance against NHS-mediated phagocytosis and killing and AMB killing. Lac1 and Lac2 are more important for age-dependent resistance against Ab-mediated killing, AMB killing, and age-dependent melanization. Furthermore, our data suggest that both Lac1 and Lac2 are needed in generationally old cells. This study thus identifies some of the key-players that contribute to age-dependent resilience of *C. neoformans*.

## Data Availability Statement

The raw data generated and analyzed in this study will be made available by the authors to any qualified researcher by request.

## Author Contributions

EO, SB, and BF contributed to the design of the study, and drafting and editing of the manuscript. MD also contributed to the reading and editing of the manuscript. EO, SB, KK, and DH contributed to acquisition and analysis of the data.

## Conflict of Interest

MD is the co-founder and Chief Scientific Officer (CSO) of MicroRid Technologies, Inc. The remaining authors declare that the research was conducted in the absence of any commercial or financial relationships that could be construed as a potential conflict of interest.
